# Dysfunctional Customer Behavior, Employee Service Sabotage, and Sustainability: Can Social Support Make a Difference?

**DOI:** 10.3390/ijerph18073628

**Published:** 2021-03-31

**Authors:** Jinsoo Hwang, Yekyoung Yoo, Insin Kim

**Affiliations:** 1 The College of Hospitality and Tourism Management, Sejong University, Seoul 143-747, Korea; jhwang@sejong.ac.kr; 2 Institute of Economics and International Trade, Pusan National University, Busan 46241, Korea; ykyoo0701@gmail.com; 3 Department of Tourism and Convention, Pusan National University, Busan 46241, Korea

**Keywords:** dysfunctional customer behavior, emotional exhaustion, service sabotage, social support, restaurant industry

## Abstract

In a restaurant industry, dysfunctional customer behavior damages customer-contact service employees’ mental health which may lead to employee defection. This study examined the effects of dysfunctional customer behavior on service employees’ service sabotage which is a mechanisms for protecting themselves from outside pressures. Additionally, it determined if emotional exhaustion plays a mediating role in the relationship between dysfunctional customer behavior and employees’ service sabotage and verified the moderating role of social support. The proposed model was tested empirically using the data from 329 restaurant customer-contact service employees in South Korea. The results indicated that dysfunctional customer behavior increased the incidence of employees’ service sabotage. Moreover, emotional exhaustion was a significant mediator in the link from dysfunctional customer behavior to employees’ service sabotage. In addition, social support moderated the effects of dysfunctional customer behavior on service sabotage. This study provides insights into the effects of dysfunctional customer behavior and methods of supporting employees socially.

## 1. Introduction

Service companies generally adopt customer-oriented service philosophies, such as the “customer is always right” ([1], p. 1795) or the “customer is king” ([2], p. 176). In the service industry based on these philosophies, the roles of customer-contact employees who deliver core services have been emphasized [3,4]; however, these employees are exposed to a variety of stressors caused by unpredictable situations [5,6,7]. According to Hu, et al. [1], approximately 82% of customer-contact employees working in the hospitality industry face violent or rude customers. In particular, violent or rude customers are encountered more frequently in restaurant outlets that supply intoxicating beverages [5]. The deviant behavior of these customers disrupts service encounters, decreases other customers’ overall service satisfaction [8,9], and damages the service companies’ financial performance [10], which is called dysfunctional customer behavior [11]. Previous service management research has found that such dysfunctional customer behavior adversely affects service employees, generating psychological stress, damaging their self-esteem, and making the service employees engage in counterproductive behaviors [6,12,13]. On the other hand, dysfunctional customer behavior in a service setting is considered a dirty little secret despite the number of known issues. Consequently, protecting the human rights of customer-contact employees in a restaurant context remains unresolved.

According to frustration-aggression theory, customer-contact service employees who experience unfair events in their workplace behave aggressively by eliciting deviant behaviors, such as service sabotage [14]. Service sabotage refers to service employees’ intentional and premeditated deviant behavior to harm a functional service encounter [15]. Although the term, sabotage, is rooted in industrial and manufacturing settings, the effects of service sabotage are far more negative on company growth and profitability compared to industrial sabotage [16]. A large number of scholars and practitioners in the service management field have paid attention to the damage caused by service sabotage because these actions harm both the service company and its customers, whereas industrial sabotage only harms the firm [16,17].

In addition to acting sabotage as a direct response to unfair events, the likelihood of service sabotage action is associated with the extent to which the service providers consider sabotage action as having potential social and emotional benefits [16]. For example, service employees enhance their self-esteem by playing pranks and entertaining coworkers who praise the saboteurs. Moreover, certain service providers restore their lost emotion by taking revenge on customers to take out their frustration. Because customer-contact service employees are exposed to diverse unpredictable situations, which corresponds to stressors, they can easily become emotionally exhausted in the workplace, which adversely affects their psychological and physical health [18]. Therefore, not only the immediate stimuli (dysfunctional customer behavior) but also the emotional state (emotional exhaustion) as predictors driving service employees’ sabotage action should be elaborately examined.

Service management researchers and practitioners highlight the importance of supporting socially customer-contact employees [19]. Although social support is a critical component to all people in any organization, it is far more beneficial to customer-contact employees in the service industry because they act as “boundary spanners”, attempting to meet the expectations of the organization and customer simultaneously [20]. Although a myriad of studies related to social support have provided evidence of a mitigating negative effect e.g., [21,22,23], the function of social support in moderating these adverse effects remains controversial. Therefore, this study examined whether social support in a service organization attenuates the detrimental effects of dysfunctional customer behavior on the employees’ negative behavior (i.e., service sabotage).

This study examined the effects of dysfunctional customer behavior in a restaurant setting on the customer-contact employees’ service sabotage, and whether employees’ emotional exhaustion is a significant mediator or not. In addition, the moderating role of social support in alleviating the negative effects of dysfunctional customer behavior on employees’ service sabotage was assessed. This study will contribute to developing restaurant service literature by providing up-to-date knowledge of the dark side of the restaurant industry by uncovering the effects of customers’ deviant behaviors and the extreme behaviors of disgruntled employees, and at the same time, offer business strategies to resolve these problems in a restaurant.

## 2. Literature Review

### 2.1. Dysfunctional Customer Behavior

Dysfunctional customer behavior refers to customer actions that disrupt service encounters by behaving against the organization’s expectations and social norms [5,24]. This behavior has been variously described by scholars using the following terms: deviant consumer behavior [25], aberrant customer behavior [26], inappropriate behavior [27], customer misbehavior [28], jay-customer behavior [9], consumer retaliation [29], unethical consumer behavior [30], customer verbal aggression [31], and customer incivility [32].

Over time, service management theorists have categorized dysfunctional customer behavior from the perspectives of the customer and employee. For example, Lovelock [33] examined such behaviors from customer viewpoints and classified them into six typologies: vandals, thieves, belligerents, family feuders, deadbeats, and rule breakers. On the other hand, Harris and Reynolds [11] examined customers’ misbehaviors from the employees’ viewpoint and classified them into eight groups by two dichotomies of motivation (i.e., covert or overt, financial or non-financial), namely compensation letter writers, undesirable customers, property abusers, service workers, vindictive customers, oral abusers, physical abusers, and sexual predators. For example, property abusers, a type of dysfunctional customer, vandalize or destroy the service firm’s items intentionally for enjoyment. Boo, et al. [34] presented six typologies of dysfunctional customer behaviors based on previous research: grungy, inconsiderate, rule breaking, crude, violent or physical abuse, and verbal abuse.

In the service management literature, one research stream of dysfunctional customer behavior focused on determining the triggers of such behaviors. For example, Reynolds and Harris [35] suggested three main drivers of dysfunctional customer behavior: personality (e.g., Machiavellianism, aggressiveness, sensation seeking, and consumer alienation), situation-specific variable (e.g., customers’ perceived inequity during service delivery), and servicescape. In addition to these triggers, Daunt and Harris [24] examined why customers misbehave, and claimed their deviant behaviors are motivated by financial gain (e.g., compensation letter writing), ego gain (e.g., sexual, verbal and property abuse to expand perpetrator’s own ego), and revenge (e.g., shoplifting and illegitimate complaining). For example, dysfunctional customers motivated by financial gain behave deliberately to gain monetary reparation post-service by complaining about the service provided without justification.

Along with the literature that examined the antecedents of dysfunctional customer behavior, consequences have also been highlighted because they cause tremendous damage during service encounters to customer-contact employees, fellow customers, and the company [5]. In particular, dysfunctional customer behavior is a financial cost to the company [10], increases employees’ psychological exhaustion and turnover [36], and decreases customer satisfaction and loyalty by disrupting other customers’ service experience [9,33].

### 2.2. Service Sabotage

Service sabotage refers to service employees’ intentional and premeditated deviant behavior to negatively influence service [15]. Over time, employees’ deviant behaviors have been defined as misbehavior [37,38], dysfunctional behavior [39], revenge [40], incivility [6], antisocial behavior [41], or counterproductive behavior [42,43]. Compared to these terms, sabotage highlights any intentional, clandestine, and purposeful behavior that is in conflict with desirable behavioral criteria and influences the service encounter negatively [15].

Historically, the concept of sabotage has attracted attention by marketing and management scholars in the manufacturing sectors because it has strong negative effects on the business performance of firms, e.g., by damaging financial profit [42,44], even endangering a company’s future performance [45]. On the other hand, recent studies e.g., [15,46,47] suggested that service sabotage could be context-specific and there are distinctive antecedents and consequences in service sectors. While industrial sabotage is limited as a protest against an organization’s injustice targeting the employees’ firm, service sabotage includes destructive behavior targeting the customers directly, which is caused by the unfair treatment of not only their company but also its customers [16,17]. Therefore, service sabotage damages the firm’s business success more than sabotage in the manufacturing sector [15].

Sabotage behaviors in the service context have been examined theoretically. For example, Harris and Ogbonna [15] categorized service sabotage into four types according to the openness of sabotage actions (covert vs. overt) and the normality of sabotage actions (routinized vs. intermittent). Employees’ service sabotage actions include jokes on customers for their own exhilaration or to entertain coworkers; negligent actions in complying with rules and regulation of the firm; adjusting the service speed by the employees’ mood or personal needs; expressions of employees’ animosity, indignation, or frustration toward the customers; delaying service by the employees’ mood and emotion; intentional inappropriate responses to customers; and taking revenge towards rude customers [2,16]. In addition, Harris and Ogbonna [46] proposed a conceptual model with seven predictors of service sabotage (i.e., risk-taking proclivity, need for social approval from coworkers, stay and pursue careers, perceived surveillance during service, perceived cultural control over service employee, the extent of contact between customers, and service employee and labor market fluidity) and five outcomes (high self-esteem, high perceived team spirit, low perceived rapport with customers, low perceived functional quality, and low perceived company performance).

According to frustration-aggression theory, frustration induces aggressive behavior [14]. Although the theory was formerly used to explain animal behavior e.g., [48], psychology scholars have recently focused on human behavior. In frustration-aggression theory, because frustration is defined as an event rather than an emotional state [49], employees who experience unfair events in service encounters behave aggressively by eliciting deviant behaviors, such as sabotage. Andersson and Pearson [50] suggested that employees who are treated unfairly are likely to reciprocate based on social exchange theory [51]. In the same vein, theorists have postulated that when customers give service employees unfair treatment, the employees might reciprocate through service sabotage [6,16]. For example, Harris and Ogbonna ([16], p. 328) showed that approximately 25% of service saboteurs are “customer revengers,” who take revenge on problem customers. Van Jaarsveld et al. [6] also revealed through their empirical study that customers’ discourteous behavior toward service employees directly influences the employees’ uncivil behavior toward customers. Thus, the following hypothesis was established:

**Hypothesis** **1** **(H1).** *Dysfunctional customer behavior increases customer-contact employees’ service sabotage.*

### 2.3. Mediating Effect of Emotional Exhaustion

As mentioned previously, emotional exhaustion refers to “*feelings of being emotionally overextended and depleted of one’s emotional resources*” ([52], pp. 20–21). Originally, the concept of emotional exhaustion was introduced as a central part of three parts in Maslach’s burnout model [53]. Although burnout is comprised of emotional exhaustion, depersonalization, which is described as “interpersonal distancing and lack of connectedness with one’s coworkers and clients,” and diminished personal accomplishment, which was described as “a negative evaluation of the self” in Maslach’s model ([54], p. 160), emotional exhaustion is considered the core component of burnout [55]. Historically, emotional exhaustion has been the focus of workplace-burnout studies because it leads to lower job satisfaction [56], higher turnover [57,58,59], less organizational citizenship behavior [60,61], and poorer job performance [62]. In particular, the emotional exhaustion of service employees is a fundamental component in generating employees’ disruptive behaviors toward their customers and organization [6,55]. Therefore, attempts to decrease the level of emotional exhaustion by service employees by thoroughly understanding it are necessary for service business success.

Theorists in the service management field suggested that customer-contact employees experience emotional exhaustion more frequently rather than other employee types [56,63]. Because customer-contact employees should play the role of a boundary spanner while interacting with customers [64], service providers require more time and energy to manage their emotions during service delivery [56]. Therefore, scholars identified the triggers of inducing service employees’ emotional exhaustion. For example, Grandey [55] claimed service employees’ surface acting requires attention and effort because of the dissonance between the inner feelings and actions, so that they deplete their cognitive and energy resources, leading to emotional exhaustion. In addition to emotional dissonance, Karatepe and Aleshinloye [65] suggested the employees’ personality also affects emotional exhaustion. Although employees’ negative affectivity leads to high emotional exhaustion, intrinsic motivation diminishes emotional exhaustion.

To service employees, dysfunctional customer behavior functions as a stressor, engendering psychological stress (e.g., a sense of shame or insult [13]). When service employees confront dysfunctional customers in their workplace, they are likely to spend time and effort handling the problem customers, which generates emotional exhaustion by draining their resources. Maslach and Jackson [66] reported that when employees in work environments with frequent contact with other people are exposed to stressors, they undergo negative psychological experiences, ultimately resulting in emotional exhaustion. Empirical studies in the service management field further support this theoretical argument. For example, Gill et al. [18] reported that a specific level of customer-contact employees’ perceived stress was related to their burnout level through employee interviews in the lodging and restaurant industries. Yagil [67] argued that dysfunctional customer behaviors, such as violent behavior and sexual abuse, lead to psychological pain, burnout, negative work attitudes, and absenteeism in service providers. In addition, Hu et al. [1] examined the effects of customer misbehaviors on cabin crew’s emotional exhaustion and the mediating functions of role stress and emotional labor in the relationship between customer misbehaviors and emotional exhaustion in the airline industry. Through an empirical field study, they found that customer misbehavior has a positive effect on the cabin crew’s emotional exhaustion, and mediators (i.e., role stress and emotional labor) play significant roles. Therefore, customer-contact employees, who have experienced dysfunctional customer behavior, face emotional exhaustion during service delivery.

Previous studies on service sabotage explained employees’ behavior by employing Hobfoll’s conservation of resources (COR) theory [68], e.g., [2], where resources are defined as “those objects, personal characteristics, conditions, or energies that are valued by the individual or that serve as a means for the attainment of these objects, personal characteristics, conditions, or energies” (p. 516). According to COR theory, when individuals encounter situations, in which they may lose physical, personal, or social resources, they make efforts to create new resources to compensate for the lost resources or minimize the resource loss [2,68]. Based on this theory, a large number of scholars [2,69,70,71,72] argued that, because service employees lose emotional resources, they invest their resources (time or energy) in practicing sabotage toward deviant customers as a way to restore and replace their lost emotions. In other words, the motivation of service employees’ sabotage is related to customer misbehavior and employee stress, so employees resort to sabotage to relieve this stress and restore exhausted emotions [47]. Service employees who feel burnt out by facing aggressive customer situations engage in surface acting [73]. In addition, if service employees continuously encounter frustrating situations, they intentionally participate in anti-social behaviors, such as sabotage [74]. Lee and Ok’s empirical study determined that service employees’ burnout is associated directly with their sabotage [2]. The likelihood of service sabotage action is associated with the extent to which the service providers consider sabotage action as having potential social and emotional benefits [16]. For example, service employees enhance their self-esteem by playing pranks to dysfunctional customers and entertaining coworkers who praise the saboteurs. Moreover, certain service providers restore their lost emotion by taking revenge on customers to take out their frustration. Thus, the following hypothesis was established:

**Hypothesis** **2** **(H2).** *Emotional exhaustion plays a mediating role in the linkage from dysfunctional customer behavior to customer-contact employees’ service sabotage.*

### 2.4. The Moderating Effect of Social Support

In general, social support is defined as the assistance provided by those who the individual is in contact with in any way to reduce the individuals’ stress levels [75,76,77]. Above all things, social support at work is considered the most important category of social support [78]. Workplace social support is described as the entire support given by the organization, supervisors, peers, and customers in a working situation [78,79]. Of the many sources of social support at work, superior support becomes a good source of restoring one’s job satisfaction [80]. Supervisor support at work encompasses emotional assistance (e.g., offering concern and empathy), informational assistance (e.g., knowledge sharing related to stressors), and instrumental assistance (e.g., resources), conveying to subordinates the critical message that they are “cared for, esteemed, and valued” ([81], p. 125). As existing organizational research identified, social support reduces the employees’ stress at work, affects job burnout, and enhances their well-being [82,83,84,85].

Although social support is an important component to all people in any organization, it is far more beneficial to customer-contact employees in the service industry. While customer-contact employees interact with both internal members (i.e., employer, supervisors, and colleagues) and external members (i.e., customers), they should act as boundary spanners, trying to meet the expectations of both parties simultaneously [20]. Therefore, customer-contact employees are often torn between being an employee in an organization and being a professional worker, consequently confronting significantly stressful circumstances [7,86]. According to the cognitive appraisal theory of stress, individuals can reduce their stress levels by gaining information on predicting potential threats or believing that they can relieve or avoid harmful situations [80]. That is, the supervisory social support that employees receive buffers the detrimental impacts of stressors on their attitudes and behavior [82].

Moreover, COR theory suggests that the individual tends to preserve one’s resources, and at the same time, accumulate resources [68]. Therefore, people with abundant resources are less sensitive to resource loss and more likely to take a risk to gain more resources [87]. In other words, people who can easily gain personal resources may waste and accumulate these resources more easily at service encounters, resulting in reduced negative outcomes [2]. For example, in the service context, customer-contact employees are required to use their physical and psychological resources when confronting dysfunctional customer behavior, ultimately inducing stress. When service employees feel that resources are insufficient for stressors, such as dysfunctional customer behavior, they engage in sabotage to restore their resources [47]. In this process, other people’s intervention to assist with stress management (i.e., supervisor support) is important [77] and helps customer-contact employees alter their perception and reactions to the stressor, thereby leading to less motivation for service sabotage [88].

The function of supervisor support in moderating the adverse effects on customer-contact employee’s sabotage behaviors can be also interpreted in view of social exchange theory. According to social exchange theory [51], the benefits received from the exchanging partner (i.e., supervisors) obligate the customer-contact employees to reciprocate with positive benefits (i.e., citizenship behaviors to the supportive supervisor). Given that an individual does not damage an exchanging partner who provides benefits due to a sense of obligation to reciprocate toward the partner, a customer-contact employee who receives high supervisory support is less susceptible to deviant behaviors at the workplace (i.e., service sabotage) despite dysfunctional customer behaviors. Previous research also suggested that the supports given by the supervisor moderate the detrimental effects of stressors at work on possible negative consequences. For example, Sakurai and Jex [21] reported that high supervisor support helps attenuate the effects of employees’ negative emotions in the workplace on the decreased work effort. The following hypothesis was derived based on the theoretical and empirical background:

**Hypothesis** **3** **(H3).** *Social support mitigates the effects of dysfunctional customer behavior on employees’ service sabotage.*

**Figure 1:**
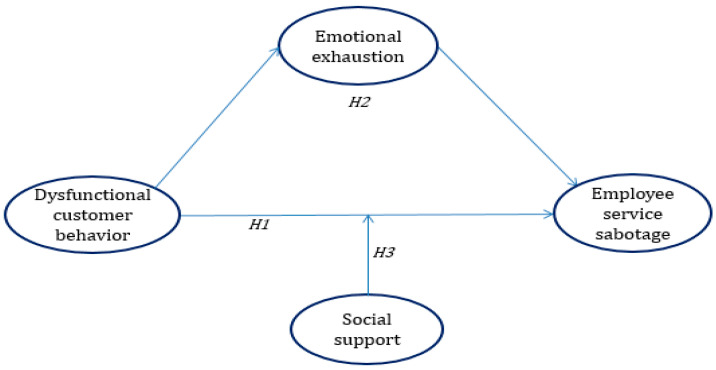
The research model.

The proposed model in the current study was presented in Figure 1.

## 3. Method

### 3.1. Measurement Items

TA quantitative approach through a field survey was employed to test the proposed model. The questionnaire for the survey was composed of three parts. The first part contained questions on customer contact-employees’ experiences, emotions, and behaviors in the workplace (i.e., dysfunctional customer behavior, emotional exhaustion, and service sabotage). The second part contained questions about what companies provide to their employees (i.e., social support). The final part comprised of demographic questions.

To measure dysfunctional customer behavior in the restaurant industry, the items were derived from the studies of Boyd [89], Harris and Reynolds [11], and Yi and Gong [13]. In addition, the items for service sabotage were derived from Harris and Ogbonna ‘s study [46]. The items for dysfunctional customer behavior and employee service sabotage were modified to better suit the restaurant industry by conducting interviews with experts and graduate students. The experts were comprised of two professors whose research fields are restaurants or hospitality, two professionals who have managed restaurants more than for five years, and three graduate students who currently work in restaurants. Of the items derived from the literature, certain scales related to dysfunctional customer behaviors and service sabotage that occur in the restaurant industry were added, and the unnecessary items were removed. The measurement items generated through this process were reviewed and pre-tested by 28 graduate students majoring in tourism and hospitality. Therefore, the awkward or unclear expressions were revised based on their feedback and the items indicating a low factor loading through a validity test were discarded. The removed items included four dysfunctional customer behavior items (“I have experienced customers who would not pay for the food service without justification”; “… customers who steal fixtures or equipment in restaurants”; “… customers making demeaning or derogatory remarks about me”; “… customers not complying with our restaurant’s regulations”) and two service sabotage items (“certain employees antagonize customers to make the rest of us laugh”; “at the restaurant outlet, I mistreat customers deliberately”). Consequently, nine items for dysfunctional customer behavior and four items for service sabotage were measured on a five-point-Likert-scale, where 1 indicates “rarely” and 5 “all the time” to represent the degree of employees’ experience. Emotional exhaustion was measured using five items adopted from Maslach and Jackson [73] and social support was measured using three items drawn from Gong et al. [90]. The above items for emotional exhaustion and social support were measured on a five-point Likert-scale, ranging from “strongly disagree” (1) to “strongly agree” (5).

### 3.2. Sampling and Data Analysis Tools

The data were collected by targeting customer-contact employees currently working at restaurants and having had experience with dysfunctional customer behavior. The questionnaires were distributed to service employees working in restaurants, and nine casual restaurants in large cities in South Korea were included, five, three and one in Seoul, Busan, and Suwon respectively. Prior to visiting the restaurants, the surveyors contacted the restaurants’ managers with the assistance of chain restaurants’ owners and asked them to approve of their customer-contact employees participating in the survey. After gaining the managers’ approval, the surveyors met the service employees without their managers to prevent any bias related to the responses of undesirable behaviors. In addition, a self-administered survey was conducted and anonymity was emphasized in the instructions of the questionnaire. From February to April 2018, we distributed approximately 30–50 questionnaires to each restaurant, for a total of 400 questionnaires. A total of 340 customer-contact employees returned their responses with complete information (85%). The quality of the 340 responses were checked by the investigators. The exclusion criteria for study participants included respondents who ticked in the only one box (e.g., all responses are 5 or 1) and repeated according to the response order (e.g., 1-2-3-4-5-1-2-3…). Through the data screening process, 11 responses were discarded. Therefore, a total of 329 responses were used for the analysis in this study.

Data analysis was conducted using the SPSS (IBM, New York, NY, USA)) and AMOS (IBM, New York, NY, USA) statistical softwares. Using frequency analysis, the characteristics of the sample were analyzed, and a normality test was performed by verifying the skewness and kurtosis value (see Appendix A). After confirming a normal distribution, the reliability and validity of the measurement variables were verified using confirmatory factor analysis (CFA). The structural relationships hypothesized in the proposed model were confirmed using structural equation modeling (SEM) analysis. A Sobel test and multiple group analysis were performed to test the mediating and moderating effects.

## 4. Results

### 4.1. Demographic Characteristics

Table 1 lists the characteristics of the 329 respondents: 214 (65.0%) females and 115 (35.0%) males. The average age was 27.7 and the majority of respondents were aged between 20 and 29 (76.8%), which reflects well that service employees in the restaurant industry in Korea are relatively young. According to National Restaurant Association [91], a large number of students get their first job in the restaurant industry, and business in the hospitality industry depends considerably on young employees [92], which resulting in a high proportion of young employees. Full-time and part-time employees accounted for 42.9% and 57.1%, respectively. Regarding the education level, bachelor’s degree holders, high school graduates, associate degree holders, and postgraduate degree holders represented 53.5%, 25.5%, 18.8%, and 2.2%, respectively. The average tenure in their current restaurant was 16 months.

### 4.2. Measurement Model Analysis

CFA was conducted to verify the reliability and validity of the latent variables and measurement items. The analysis results of the measurement model indicated that the fit indices were all within the acceptable levels (χ^2^ = 448.354, df = 177, χ^2^/df = 2.759, *p* < 0.001). In addition, comparative fit index (CFI) = 0.904 and incremental fit index (IFI) = 0.905 exceeded the criterion value of 0.900. Root mean square error of approximation (RMSEA) was 0.073, indicating below the criteria value of 0.08. As shown in Table 2, all measurement items were well loaded on each construct, showing substantial factor loadings between 0.553 and 0.936 (*p* < 0.001). The average variance extracted (AVE) values for all the latent constructs exceeded 0.5, as shown in Table 3, which ensures that convergent validity was confirmed [93].

To evaluate the discriminant validity of each construct concept, the squared value of the correlation coefficient of each concept was compared with the AVE value [94]. The discriminant validity was satisfied because the squared correlation coefficients of the three pairs of constructs were all below the minimum AVE value of constructs. Furthermore, as the construct reliability (CR) values were over the cut-off of 0.7, all factors were confirmed to be reliable concepts with high internal consistency [93].

### 4.3. Hypotheses Test

SEM was used to verify the causal relationships between the constructs hypothesized in the research model. The structural model fits the data well, showing substantial goodness-of-fit indices (χ^2^ = 361.222, df = 126, χ^2^/df = 2.867, *p* < 0.001, CFI = 0.909, IFI = 0.910, RMSEA = 0.075). Structural model analysis results showed that dysfunctional customer behavior had a positive direct effect on the employees’ service sabotage (β = 0.512, t = 5.414), supporting H1.

To verify the mediation effect of emotional exhaustion in the relationship between dysfunctional customer behavior and employees’ service sabotage, 5000 resamples were gathered by utilizing bias-corrected bootstrap 95% confidence intervals [95]. The results showed that the indirect effect is significant (β _dysfunctional customer behavior__→__emotional exhaustion →_ _service sabotage_ = 0.063, *p* < 0.05). Additionally, to verify if emotional exhaustion is full or partial mediator, the path from emotional exhaustion to service sabotage was constrained to zero. Using chi-square difference test, chi-square difference between free model and constrained model was significant (Δχ^2^= 5.076, Δdf = 1) at *p* < 0.05. The impact of dysfunctional customer behavior on service sabotage was higher in a constrained model (β = 0.616, *t* = 6.450) than an unconstrained model (β = 0.512, t = 5.414), showing that the relationship between dysfunctional customer behavior and employee service sabotage is partially mediated through emotional exhaustion. Accordingly, H2 is supported.

### 4.4. Moderating Effect Test

Multiple group analysis was conducted to verify the moderating function of social support in the relationships between dysfunctional customer behavior and service sabotage (H3). For multiple group analysis, the sample was dichotomized into two groups (high social support-group, n = 150 vs. low social support-group, n = 179) using the median split based on either above or below the median value of the sum of three items indicating social support. The chi-square value difference was compared according to the degree of freedom difference between the unconstrained and constrained models [96].

A significant chi-square difference was observed across the two groups regarding the effect of dysfunctional customer behavior on service employees’ sabotage (Δχ^2^ = 5.004 > χ^2^_0.05_(1) = 3.84, df = 1). In particular, dysfunctional customer behavior generated employees’ service sabotage in the group of low social support, whereas it did not have an effect on employees’ service sabotage in the high social support group (low: β = 0.768, *p* < 0.05 vs. high: β = 0.146, *p* > 0.05). That is, H3 was supported (see Table 4).

Table 5 illustrates the analysis results of the hypothesized model.

## 5. Discussion and Implications

The research objectives were as follows: (1) to examine the effects of dysfunctional customer behavior on customer-contact employees’ service sabotage in a restaurant context; (2) to determine if the employees’ emotional exhaustion mediates the effect of dysfunctional customer behavior on employees’ service sabotage; and (3) to verify the moderating function of social support in alleviating the negative effects of dysfunctional customer behavior on employee service sabotage. The conceptual model was tested through an empirical field survey, and the analysis results provided the following theoretical and practical implications.

First, dysfunctional customer behavior plays a crucial role in inducing employees’ service sabotage actions in a restaurant context. According to marketing and psychological theories, unfair events (i.e., frustration) provoke aggressive behavior (frustration-aggression theory [14]), and individuals treated unjustly are likely to reciprocate their counterpart’s actions (social exchange theory [51]). This study is significant in that these theories were tested empirically through a field study on restaurant customer-contact employees, indicating the applicability of these theories to the restaurant service industry.

Employees devise a variety of service sabotage, with more than 85% of employees having sabotaged their service encounters [16]. In particular, sabotage in the service industry is caused by customers’ misbehavior, unlike in the manufacturing industry. Individuals instinctually protect themselves from outside pressures and there is also a tendency to retaliate (i.e., the tendency of equity), as argued in social exchange theory. Therefore, service companies, including restaurants, should not only treat their employees fairly in the workplace (e.g., determining the fairness of promotion methods), but also help them protect themselves from inappropriate customer behavior. In addition, restaurant managers need to devise strategies to decrease the motivation of a customer’s deviant behavior. For example, an event, such as “using courteous or comforting words”, can be conducted during the slow times of the day, such as weekday lunch hours. In particular, customers who politely greet the customer-contact employees or treat the employees with well-mannered behavior can receive a price discount or a free drink, thereby developing customer-employee rapports [97].

Second, the emotional exhaustion of customer-contact employees significantly mediates the relationship between dysfunctional customer behavior and employee service sabotage in a restaurant context. According to marketing and organizational literature, because dysfunctional customer behavior becomes a harmful stressor to service employees [67,91,98], they are emotionally exhausted when confronting dysfunctional customer behavior [1]. In addition, human resource management theorists have suggested that when individuals lose emotional resources, they make efforts to restore resources to compensate for the lost resources or minimize resource loss (COR theory [68]), thereby generating employees’ service sabotage [2]. Thus, this study expanded this reasoning to the restaurant industry by empirically confirming existing arguments. The current study findings suggest that restaurant executives and managers should examine and identify the types of dysfunctional customer behavior thoroughly and develop manuals that explain how to handle dysfunctional customer behavior according to the types, leading to less emotional exhaustion.

Third, the significant moderating role of social support was identified in this study. The results of the data analysis showed that dysfunctional customer behavior induced employee service sabotage in the group with low social support, whereas it did not influence employee service sabotage in the group with high social support. This can be interpreted as follows. Service employees with low social support try to restore lost resources themselves by sabotage, whereas employees with high social support tend to readily use their resources because they can acquire personal resources more easily when faced with a dysfunctional customer. That is, when customer-contact employees feel socially supported by significant people, such as their supervisors or managers in the restaurant, they can treat the customer’s misbehavior more appropriately. The finding is significant in that the current study confirmed empirically the theory that people with abundant resources are less sensitive to resource loss [87] in a restaurant context.

This result has crucial implications for restaurant owners and managers. Restaurants should use distinctive tactics to make customer-contact employees feel socially supported. For example, Southwest Airlines has the philosophy that “employees always come first” rather than customers ([99], p. 538). When Herb Kelleher, the former CEO of Southwest Airline, received a complaint letter from a disgruntled customer, he replied with “we will miss you!” to the letter instead of apologizing or chastising the flight attendant [100]. The type of social support that Southwest Airlines provides makes customer-contact employees perceive justice by appropriate intervention, leading to employees’ favorable attitudes and behaviors [101] (i.e., less service sabotage [6]). In addition, restaurant owners should train supervisors on how to provide social support to customer-contact service employees to relieve tension. For example, owners need to motivate immediate supervisors to share tacit or uncodified knowledge built through the experience of dysfunctional customer behaviors to their customer-contact employees. By doing so, the customer-contact employees can flexibly cope with difficult situations caused by dysfunctional customer behaviors rather than resorting to service sabotage.

One of the most important implications of this study is that social support was introduced as a moderating variable based on COR theory, which corresponds to the active efforts of service companies to decrease service sabotage by customer-contact employees. This is different from other concepts proposed in previous research, such as emotional intelligence [2], which is related to the individual characteristics according to which employees control their own emotions and behavior. Therefore, this study contributes academically to developing a body of knowledge in the restaurant industry.

Despite the implications of the current study, there was one limitation. Although the concept of employees’ service sabotage is also associated with inequity caused by the company, this study was limited to unfairness generated by the customers (i.e., dysfunctional customer behavior). Service sabotage is a behavior that can be triggered not only by the customers but also by other social causes related to the individual or organization. Therefore, future research should introduce the factors that induce the employees’ inequity perception by caused by customers as well as by the organization (e.g., wage or promotion), and need to identify what drivers are more powerful. Additionally, the current study collected information from customer-contact service employees using self-report method, which could generate social desirability response bias. In the future research, data gained from supervisor-subordinate dyads need to be used to prevent the social desirability response bias. Another issue of our sample is limited to the casual restaurant in big cities, therefore area for data collection should be expanded. Finally, even though social support used in this study focused on support by supervisor who directly related to the customer-contact service employees, it is needed to expand to social support from leaders for identifying social exchange between leaders and employees.

## Figures and Tables

**Table 1:** Profile of respondents.

Characteristics	Categories	Frequency (N)	Percentage (%)
Gender	Male	115	35.0
	Female	214	65.0
Age (Mean = 27.7 years old)	20s	253	76.8
	30s	54	16.4
	40s	12	3.7
	Older than 50s	10	3.0
Type of employment	Full-time	141	42.9
	Part-time	188	57.1
Education level	High school diploma	84	25.5
	Associate’s degree	62	18.9
	Bachelor’s degree	176	53.5
	Graduate degree	7	2.1
Average work period: 16 months			

**Table 2:** Confirmatory factor analysis: Items and loadings.

Items	Loading	Cronbach’s α
Dysfunctional customer behavior		0.813
I have experienced verbal abuse from customers in our restaurant	0.778	
customers putting me down or being condescending to me	0.859	
customers always complaining about us	0.553	
customers making offensive sexual comments to me	0.850	
customers making negative or obscene gestures to me	0.885	
customers physical abuse from customers in our restaurant	0.595	
customers being drunk and disorderly conduct in our restaurant	0.709	
customers taking out their own frustrations on me	0.874	
customers staring, making dirty looks or negative eye-contact	0.594	
Emotional exhaustion		0.853
I feel fatigued when I get up in the morning and have to face another day on the job	0.611	
I feel emotionally drained from my work	0.712	
Working with people all day is really a strain for me	0.850	
I feel like I am at the end of my rope	0.719	
Working directly with people places too much stress on me	0.678	
Service sabotage		0.826
I take revenge on rude customers	0.869	
I slow down service when I want to	0.665	
When customers are not looking, I deliberately mess things up	0.769	
I ignore the restaurant’s service rules to make things easier	0.744	

Notes: All factor loadings were significant at *p* < 0.001.

**Table 3:** Descriptive statistics and associated measures.

Construct		Mean	SD	CR	AVE	1	2	3	4
1	Dysfunctional customer behavior	2.39	0.501	0.949	0.570	1.00			
2	Emotional exhaustion	3.01	0.705	0.871	0.515	0.389 (0.151)	1.00		
3	Service sabotage	2.48	0.761	0.866	0.586	0.571 (0.326)	0.362 (0.131)	1.00	
4	Social support	3.59	0.696	0.943	0.728	−0.026 (0.001)	−0.350 (0.123)	−0.117 (0.013)	1.00

Note: SD = standard deviation; CR = composite reliability; AVE = Average variance extracted; squared correlations are presented in parentheses.

**Table 4:** Moderating test results.

Paths	High Social Support Group (n = 150)		Low Social Support Group (n = 179)	
	Standardized Estimate	T-Value	Standardized Estimate	T-Value
Dysfunctional customer behavior → service sabotage	0.146	1.503	0.768 **	5.735
**Chi-Square Difference Test**	**Baseline Model**		**Restricted Model**	
Chi-square (df)	χ^2^(252) = 536.261		χ^2^(253) = 541.265	
Result: △χ^2^(1) = 5.004, *p* < 0.05 (Significant)				

Note: ** *p* < 0.01.

**Table 5:** Hypotheses test results.

Hypotheses		Result	Support
H1	Dysfunctional customer behavior → Service sabotage		
	Standardized estimate: 0.512 **; *t*-value: 5.414	Significant path	Yes
H2	Emotional exhaustion’s mediating effect in the link from dysfunctional customer behavior and service sabotage		
	Indirect effect: β _dysfunctional customer behavior__→__emotional exhaustion__→__service sabotage_ = 0.063 *	Partial mediator	Yes
	Total effect: β _dysfunctional customer behavior__→__emotional exhaustion__→__service sabotage_ = 0.575 **		
H3	Social support’s moderating effect on the relationship between dysfunctional customer behavior and service sabotage	Significant moderator	Yes

Note: * *p* < 0.05, ** *p* < 0.01.

## Data Availability

Data available on request.

## Appendix A

**Table A1:** Normal distribution tests.

Constructs	Items	Mean	Standard Deviation	Skewness	Kurtosis
Dysfunctional customer behavior	DCB1	3.31	0.769	0.059	−0.209
	DCB2	3.18	0.866	−0.037	−0.576
	DCB3	2.68	0.836	−0.129	−0.110
	DCB4	1.75	0.736	0.800	0.470
	DCB5	1.58	0.681	1.055	1.025
	DCB6	1.62	0.731	0.956	0.339
	DCB7	3.00	0.843	−0.455	−0.050
	DCB8	2.54	0.900	−0.006	−0.652
	DCB9	1.85	0.737	0.524	−0.144
Emotional exhaustion	EX1	3.56	0.843	−0.485	0.126
	EX2	3.07	0.856	0.018	−0.080
	EX3	2.93	0.912	0.108	−0.123
	EX4	2.71	0.930	0.262	−0.161
	EX5	2.78	0.899	0.231	0.151
Service sabotage	SSA1	2.33	0.921	0.252	−0.744
	SSA2	2.17	0.844	0.348	−0.449
	SSA3	2.62	1.024	0.084	−0.869
	SSA4	2.81	0.957	−0.35	−0.745
Social support	SOST1	3.66	0.677	−0.643	1.429
	SOST2	3.53	0.703	−0.315	0.434
	SOST3	3.58	0.708	−0.381	0.219
